# Identification of the volatile profiles of 22 traditional and newly bred maize varieties and their porridges by PTR‐QiTOF‐MS and HS‐SPME GC–MS

**DOI:** 10.1002/jsfa.10781

**Published:** 2020-09-21

**Authors:** Onu Ekpa, Vincenzo Fogliano, Anita Linnemann

**Affiliations:** ^1^ Food Quality and Design Group, Department of Agrotechnology and Food Sciences Wageningen University and Research Centre Wageningen The Netherlands

**Keywords:** volatile aroma compounds, maize, porridge, PTR‐MS, GC–MS, Africa

## Abstract

**BACKGROUND:**

Low adoption of maize varieties bred to address the nutritional needs of the growing African population limits their impact. Aroma is essential in consumer preference, but has hitherto hardly been studied. We analysed the volatile organic compounds of flours and porridges of 22 maize varieties belonging to four nutritionally distinct groups, namely provitamin A maize, quality protein maize, yellow and white maize.

**RESULTS:**

Proton‐transfer‐reaction quadrupole ion time‐of‐flight mass spectrometry (PTR‐QiTOF‐MS) analysis generated 524 mass peaks ranging from 16.007 to 448.089 *m/z*. Principal component analysis separated the varieties belonging to the four groups. With headspace solid‐phase microextraction gas chromatography–mass spectrometry (HS‐SPME GC–MS), 48 volatile compounds were identified in maize flour and 21 in maize porridge, including hexane, nonane, pentanoic acid, 1‐octen‐3‐ol, 1‐hexanol, hexanal, nonanal, 2‐pentylfuran and 2‐heptanone. Volatile compounds such as 1,2,4‐trimethyl benzene, associated with thermal degradation of carotenoids, increased in the porridge of yellow and provitamin A maize.

**CONCLUSION:**

The results indicate that PTR‐QiTOF‐MS and HS‐SPME GC–MS combined with multivariate analysis are instrumental to study the volatile aroma compounds of different maize varieties.

## INTRODUCTION

Several maize varieties have been bred to cater for the rising food demand (i.e. high yield, drought and pest‐resistant varieties) and nutritional deficiencies (i.e. quality protein, provitamin A, zinc and iron biofortified varieties) in Africa.^1^ However, low acceptance and adoption of these improved varieties persist, resulting in a limited impact on the target population. Various reasons have been proposed to explain low adoption.^2^ Of particular interest is the sensory quality: Africans have shown a high preference for the flavour of white maize and their traditional varieties.^1^ Considering that flavour properties can significantly affect consumer preferences, one would expect the aroma profile of maize to have been thoroughly studied and reported. Surprisingly, this is not the case: very few and old publications are available.^3^, ^4^, ^5^, ^6^

Volatile compounds of other cereals such as rice, wheat and recently millet have been widely studied.^7^ Extensive volatile organic compound (VOC) studies on rice have facilitated a significant improvement of its taste and acceptability.^8^ A particular point of concern regarding maize is that some biofortified maize varieties, such as the yellow and orange, are rich in oils and carotenoids. Therefore they are prone to lipid spoilage, especially under tropical conditions, which could result in off‐flavour formation during processing and storage.^9^ In this article, the volatile compounds determining the aroma of maize were assessed by headspace analysis with a proton‐transfer‐reaction quadrupole ion time‐of‐flight mass spectrometry (PTR‐QiTOF‐MS). This innovative instrument enables direct injection of headspace volatiles without any need for pre‐treatment or chemical extraction and has a high resolution and sensitivity, thereby allowing high‐speed data collection and detection of trace compounds that are essential for aroma profiling.^10^ Such sophisticated and high‐resolution equipment is crucial because maize is characterized by low levels of aroma compounds.^11^ However, the PTR‐QiTOF‐MS technique is not sufficient to give a confirmatory identification of VOCs, hence headspace solid‐phase microextraction gas chromatography–mass spectrometry (HS‐SPME GC–MS) was also used.

In the present study, PTR‐QiTOF‐MS was used for the first time on maize with the aim of identifying the volatile profile or fingerprint of different varieties and their porridges. We opted for porridges as these are the most common foods prepared from maize flour throughout Africa.^12^ Knowledge about maize aroma could help breeders to fine‐tune improved varieties to meet users’ preferences, and thus develop maize varieties with appreciated flavours, which will improve consumer acceptability. Hence the main objective of this research was to determine the volatile profiles of flour from 22 commonly used maize varieties, i.e. quality protein maize (QPM), provitamin A biofortified maize, white and yellow maize, as well as their porridges.

## MATERIALS AND METHODS

### Maize flour, porridge preparation and reagents

Twenty‐two maize varieties were obtained from the International Institute of Tropical Agriculture (IITA), Nigeria, and the Centre de Recherches Agricoles, Benin (INRAB), Supporting Information, Table S1. The samples were stored at 4 °C away from light and in airproof bags to prevent oxidation and loss of volatiles. The moisture content was determined according to AACC method 44‐15‐02. Maize porridge was prepared by slowly adding boiling water to 30% maize flour under continuous stirring for 10 min. Next, the porridge was covered with aluminium foil and allowed to stand for 5 min.^13^

The following standards were used for identification of VOCs by GC–MS: C7‐C40 saturated alkanes (multi‐component solution with 34 analytes), heptanoic acid, 2‐propanol, 2‐octanol, 1‐heptanol, acetaldehyde, limonene, 2‐mercaptoethanol, benzaldehyde, hexanal, 2‐butanol, ethanol, benzene, dimethyl sulfoxide, 1‐octanol, acetonitrile, 2‐propanol, *n*‐hexane and pentane. All standards had purity higher than 99% and were purchased from Merck (Darmstadt, Germany) and Actu‐All Chemicals (Oss, the Netherlands).

### PTR‐QiTOF‐MS analysis

Briefly, 3 g of maize sample in 250 mL glass bottle was agitated at 46 g for 30 min at 40 °C. The headspace of the sample was analysed by connecting the bottle to the inlet flow of the PTR‐QiTOF‐MS/MS 8000 instrument (Ionicon Analytic GmbH, Innsbruck, Austria). The mass scale was calibrated using the peaks of recognized components, namely the NO^+^ peak, *m/z* = 29.9974 and acetone, C_3_H_7_O^+^, *m/z* = 59.0497, to guarantee high mass accuracy throughout the analysis. The instrumental conditions for the proton transfer were: a drift voltage of 650 V, drift temperature of 60 °C, drift pressure of 3.80 mbar, and an *E/N* (electric field strength/particle density number) value of 120 Td (1 Td = 10^−17^ cm^2^ V^−1^ s^−1^). Sampling was at a flow rate of 50 mL min^‐1^. Every sample measurement started with flushing the PTR machine with ambient air of the bottle for 10 s as the blank. Then, the sample was measured for 40 s, followed by flushing for 10 s.

### HS‐SPME GC–MS analysis

Samples were analysed using stabilwax DA capillary column (30 m × 0.25 mm ID × 0.25 μm) and SPME fibre assembly DVB/CAR/PDMS (Supelco, Bellefonte, PA, USA). Calibration of HS‐SPME GC–MS mass scale was regularly performed using perfluorotributylamine (PFTBA). Briefly, 2 g of the maize flour was put in a 10 mL glass vial, crimped, and incubated at 40 °C for 15 min. This was followed by 10 min of headspace absorption and another 10 min desorption in the GC. The oven temperature for SPME injection was 40 °C for 2 min, increased at 10 °C min^−1^ to 200 °C and then held at 200 °C for 5 min. The carrier gas was helium at a flow rate of 1 mL min^−1^.

### Data processing and statistical analysis

The raw PTR‐QiTOF‐MS data were processed using PTRwid.^14^ The data was blank corrected using RStudio 1.1.383 (the R Foundation for Statistical Computing, Vienna, Austria) and all ions interfering (especially water clusters) were manually removed. Integrated peaks signals from PTR‐QiTOF‐MS in units of cps (corresponding to the mass spectral intensity) were used for further analysis.

Chromeleon 7.2 (Thermo Fisher Scientific Inc., Waltham, MA, USA) was used to analyse the GC–MS data. The VOCs detected were identified by matching their mass peaks with the NIST spectral library database, the retention indexes according to literature and C7–C40 saturated alkanes retention index marker probe. When available, the MS identifications were confirmed by matching the GC retention times of the analytes with pure standards.

Non‐parametric tests (Kruskall Wallis test and Dunn's *post hoc* test) were performed using IBM SPSS® software version 23 since the experimental groups had different sample sizes, unequal variances and the data were not normally distributed (Shapiro–Wilk test < 0.05).^15^ For both porridge and flour data, principal component analysis (PCA) was performed using XLSTAT® data analysis software (version 2018.5.52280, Addinsoft, New York, NY, USA) for Microsoft Excel®. The significance level was fixed at *P* < 0.05.

## RESULTS AND DISCUSSION

### Detection of volatile compounds in maize flour and porridge by PTR‐QiTOF‐MS

**Figure 1:**
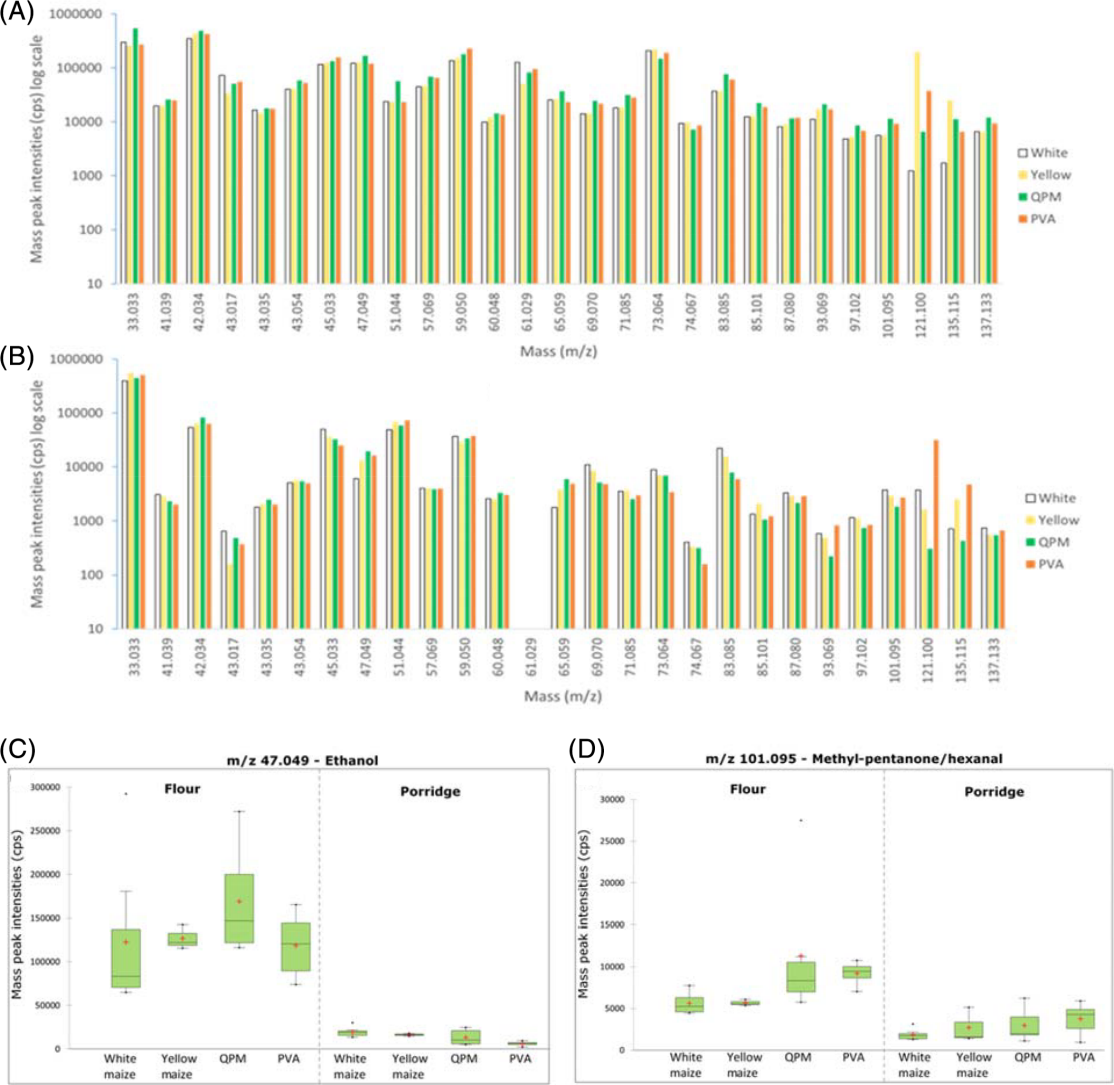
Mean mass peak intensities of the dominant ions (A) maize flour and (B) maize porridge in the headspace generated by PTR‐QiTOF‐MS. Box plot of some selected mass peaks generated by PTR‐QiTOF‐MS significantly different in the maize samples (the mass peaks tentatively identified as shown on the charts). ‘+’ indicates mean point and ‘.’ indicates minimum/maximum point.

The analysis of maize samples (flour and porridge) generated 524 mass peaks in the range of *m/z* 16.007 to 448.089. The average blank corrected masses in count per seconds (cps) served as a means of comparison between each sample and group. An example of the predominant mean mass peaks in the four groups is shown in Fig. 1. White maize group recorded the lowest peaks in maize flour but showed higher peak of VOCs in the porridge. Compounds having masses such as *m/z* 33.030, 38.034, 51.044, 127.112 and 143.143 increased in all porridges. However, a compound having a mass of *m/z* 61.029 was lost in the porridge of all varieties, possibly due to high volatility, fast thermal degradation or hydrolysis in contact with hot water (Fig. 1). For instance, *m/z* 41.039, 43.017, 43.054, 57.069, 58.072, 73.064, 74.067, 85.101, 89.059, 93.069 and 137.133 were reduced up to 90% in all the samples (Table S2). The mass peaks of the porridges show degradation of many masses that were abundant in the maize flour. Buttery *et al*.^3^ observed that volatiles found in uncooked maize were below detection in cooked maize due to steam vaporization during cooking. Some lower stable masses in porridge possibly existed as a thermal degradation fragment of higher masses. Correlation matrix (Spearman) showed that lower molecular weight ions are negatively correlated to the higher molecular weight ions in porridge, whereas in flour the ions are positively correlated (Table S3).

Multiple pairwise comparisons of masses using Dunn's procedure showed that white and provitamin A (PVA) maize flours were significantly different (Table S2). White maize and QPM are significantly differentiated by *m/z* 54.034, 97.064, 98.068, 99.080, 100.044, 100.081, 112.047, 129.127 and 429.089. The yellow and PVA maize flours, which are closely related in terms of colour and carotenoid content, were shown to be significantly different in the measured mass, 84.185 and 256.780, respectively. A similar trend was observed in maize porridges wherein 43 masses significantly differentiated white maize and PVA maize flour (Table S2). In contrast to the result of maize flour, *m/z* 80.047, 95.047, 123.044, 152.148 and 193.16 significantly distinguished white maize from yellow maize porridge. In general, there is an abundance of masses that differentiate white maize from PVA maize compared to other pairwise comparisons. Differences in volatile composition and abundance were observed among the maize varieties within the same group (Fig. 1(box plot)). In PVA porridge, for instance, variety A4 recorded the lowest peak intensity (1791.53 cps) while variety A5 in the same group had a peak intensity of 4352.82 cps for *m/z* 83.049 – methyl‐furan. Such diversity within the same group creates an opportunity for future improvement of maize varieties.

As shown in Table 1, the most abundant mass peaks of maize flour and porridge detected by PTR‐QiTOF‐MS were *m/z* 33.033, 42.034, 43.017, 45.033, 47.049, 49.011, 51.044, 59.050, 61.029 and 135.115. These masses, tentatively identified as methanol, acetonitrile, acetaldehyde, ethanol, methanethiol, acetone/propanal, acetic acid and terpene, respectively, have been associated with maize through conventional GC–MS analysis.^3^, ^4^, ^5^, ^16^, ^17^ In fact, Flora *et al*.^16^ identified methanethiol, acetaldehyde, ethanol and acetone as the predominant peaks responsible for the fruity and sulphurous aroma of sweet corn. Likewise, Gonçalves *et al*.^11^ found β‐myrcene (molecular weight 136.23; monoterpene) to be abundant in maize flour. Terpene compounds of similar masses such as limonene and β‐ocimene have been found in maize by other researchers.^5^, ^11^

**Table 1:** Average predominant mass peak intensities extracted by proton‐transfer‐reaction quadrupole ion time‐of‐flight mass spectrometry (PTR‐QiTOF‐MS) in the headspace of maize flour and porridge – white maize, yellow maize, quality protein maize (QPM) and provitamin A (PVA) maize, with *P*‐values where significant differences are indicated by superscript letters (Kruskal–Wallis test; Dunn's test, *P* < 0.05)

Measured mass, *m/z*	33.033	42.034	43.017	45.033	47.049	49.011	51.044	59.050	61.029	135.115
*Maize flour: average mass peak intensities (log, cps)*										
White maize	5.47^a^	5.54^a^	4.86^a^	5.06^a^	5.09^a^	3.51^a^	4.37^a^	5.13^a^	5.10^a^	3.23^a^
Yellow maize	5.41^a^	5.64^a^	4.53^a^	5.09^a^	5.10^a^	3.47^a^	4.37^a^	5.19^ab^	4.71^a^	4.39^a^
QPM	5.73^a^	5.69^a^	4.71^a^	5.12^a^	5.23^a^	3.54^a^	4.75^a^	5.25^ab^	4.92^a^	4.04^a^
PVA maize	5.43^a^	5.63^a^	4.75^a^	5.19^a^	5.07^a^	3.49^a^	4.38^a^	5.35^b^	4.98^a^	3.82^a^
*P*‐Value	0.30	0.33	0.56	0.24	0.30	0.68	0.65	0.01	0.37	0.11
*Maize porridge: average mass peak intensities (log, cps)*										
White maize	5.65^a^	4.92^a^	2.83^a^	4.51^a^	4.29^a^	3.93^a^	4.77^a^	4.53^a^	ND	2.63^a^
Yellow maize	5.70^a^	4.80^a^	3.05^a^	4.39^a^	4.21^ab^	4.02^a^	4.86^a^	4.57^a^	ND	3.68^a^
QPM	5.70^a^	4.81^a^	3.00^a^	4.56^a^	4.12^ab^	4.10^a^	4.84^a^	4.47^a^	ND	3.41^a^
PVA maize	5.59^a^	4.73^a^	2.89^a^	4.70^a^	3.79^b^	4.27^a^	4.69^a^	4.56^a^	ND	2.86^a^
*P*‐Value	0.44	0.05^a^	0.29^a^	0.17	0.02	0.08	0.37	0.62	—	0.52
Tentative identification	Methanol	Acetonitrile	Fragment (diverse origin)	Acetaldehyde	Ethanol	Methanethiol	NI	Acetone/propanal	Acetic acid	Terpene (*unknown*)
Sum formula	CH_5_O^+^	C_2_H_4_N^+^	C_2_H_3_O^+^	C_2_H_5_0^+^	C_2_H_5_OH^+^	CH_4_SH^+^	—	C_3_H_6_OH^+^	C_2_H_4_O_2_H^+^	C_10_H_15_^+^
References	10, 30, 31, 32	31, 34	34	30, 31, 34	10	10, 32	—	10	10	34, 35

ND, not detected; NI, not identified.

Superscripts with different letters indicate significant differences between the groups (*p* < 0.05).

Generally, the amount of the ten predominant masses decreased in the porridge, except for *m/z* 33.033 and 51.044, which showed stability and a significant increase in all varieties except the QPM. The *m/z* 61.029 was abundant in maize flour but not present in the porridge. This compound could have been lost by thermal degradation and vaporization. Volatiles present in raw maize and below detection in cooked maize, are likely lost due to vaporization.^3^ Compound found in porridge such as *m/z* 49.011, methanethiol (otherwise known as methyl mercaptan), was generated during porridge preparation, possibly due to heat treatment and/or hydration processes. In combination with other VOCs, methanethiol is an important aroma contributor to cooked cereals, e.g. cooked rice.^8^ Overall, only *m/z* 59.050 showed a significant difference (*P*‐value = 0.01) in the ten predominant masses in the four groups, while *m/z* 42.034 and 47.049 were significantly different in porridges at *P*‐values = 0.05 and 0.02, respectively (Table 1). Other masses with significant differences (*P* < 0.05) but not dominant are shown in (Table S2).

### Clustering of the volatiles of the four groups of maize varieties

**Figure 2:**
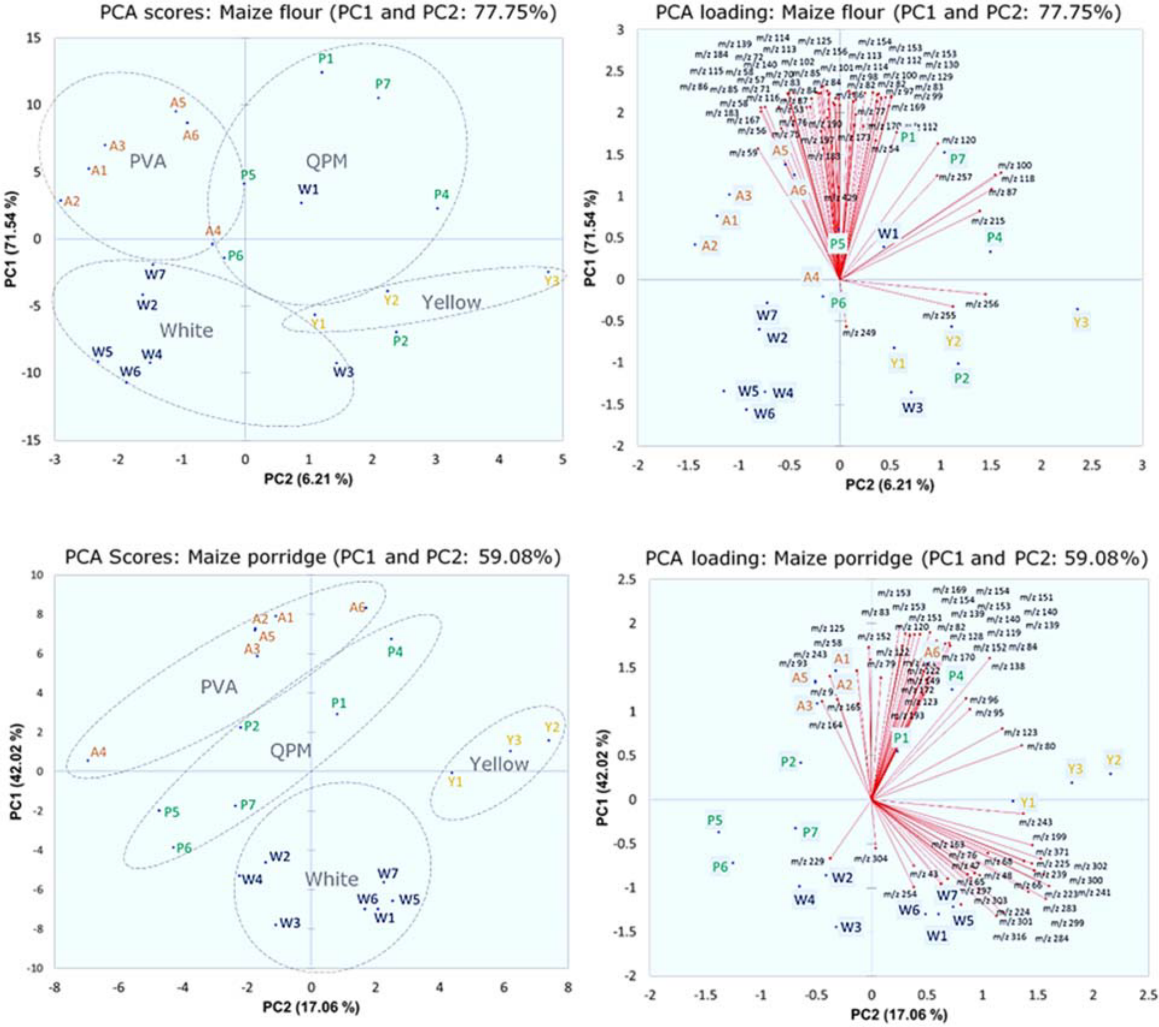
Plots of two dimensions of PCA on the mass spectral data of VOCs in maize flour and porridge of the PTR‐QiTOF‐MS data. The plots were derived from 66 and 69 mass peaks extracted from maize flour and porridge, respectively. These masses showed significant difference (*P* < 0.05, Kruskal–Wallis test and Dunn's test) in all varieties, thus being useful for PCA cluster formation. White maize (blue), provitamin A (PVA) maize (orange), yellow maize (yellow) and quality protein maize (QPM) (green) are separated in the PCA plot.

Kruskal–Wallis test and Dunn's test as *post hoc* analysis of the 524 mass peaks generated by PTR‐QiTOF‐MS indicated 66 significantly different mass peaks for maize flour and 69 for maize porridge (*P* < 0.05) (Table S2). All significantly different mass peaks were subjected to PCA to identify differences between the 22 maize varieties. A PCA score plot is shown in Fig. 2, which indicates the grouping of the maize samples for the four groups.

The first two principal components (PCs) for maize flour explain 77.75% variance. All maize flour samples (except the PVA maize) are more spread out as compared to the maize porridge. The QPM varieties are located in between the other three groups, although the distribution is dispersed. Overlap is between the region of QPM and others, suggesting that the groups are more diverse in their volatile profiles. This is not strange because QPM varieties are usually developed from either the white or yellow maize. In fact, QPM (P2) and (P4) located close to the yellow maize were identified as the yellowish 2000 EVDT Y STR QPM and 2009 TZE OR2 DT STR QPM, respectively (Fig. 2). Similarly, QPM variety (P6) located in close proximity with the white maize group was identified as white DMR ESR/QPM (Table S1). The loading plots corresponding to the PC1 *versus* PC2 are shown in Fig. 2(right), indicating masses that are important to each cluster.

The first two PCs for maize porridge explain 59.08% variance – much lower than for maize flour, see Fig. 2. Although all varieties are well separated in the PCA, the lower explained variance shows the complexity of the differences between the varieties after porridge preparation. QPM varieties are well separated in the PCA, the samples were spread out and formed a borderline between all varietal groups, as seen in maize flour PCA. Interestingly, all QPM varieties that are white (P5, P6 and P7) tilted towards white maize group while those that are yellow (P1, P2 and P4) drifted towards yellow/orange groups. As shown in the PCA plot, a large difference exists in the volatile profiles of white maize and the PVA maize. Concerning the PCA plot, our preliminary run using Pirouette® Infometrix software based on mean centred and normalized scale pre‐processing method resulted in good separation and explained more than 90% (PC1 and PC2) of the total variability in the samples. However, the current research adopted a method with minimal preprocessing of data, XLSTAT, to maintain data as close as possible to their raw and reproducible form. Considering the pattern of the PCA, it can be resolved that the distinguishing VOCs of the maize varieties were found with the help of PTR‐QiTOF‐MS. Hence this rapid and direct technique can be used for characterization of different maize varieties.

### Composition of volatiles in maize flour and porridge assessed by GC–MS

GC–MS is the conventional method for volatile analysis in food matrices due to its reliable compound identification and quantification. However, PTR‐MS equipment is rapid and sensitive but difficult for compound identification even with the advent of the new versions with TOF.^18^ Therefore, the identification of compounds with PTR techniques remain tentative and depend on an in‐depth understanding of volatiles in the specific food matrix. However, PTR‐MS techniques have shown to be effective in fingerprinting or ‘quick scan’ and classification of samples, thus the current work focused on this important ability of the instrument, while GC–MS was used for compound identification. A one‐on‐one link between the two instruments has not been established.^18^, ^19^ Furthermore, an alternative method to static headspace volatile detection used in the current work is simultaneous distillation extraction (SDE) for sample preparation prior to GC, but extraction method may not signify actual volatile production in the flour or porridge under tropical or cooking conditions.^7^

**Figure 3:**
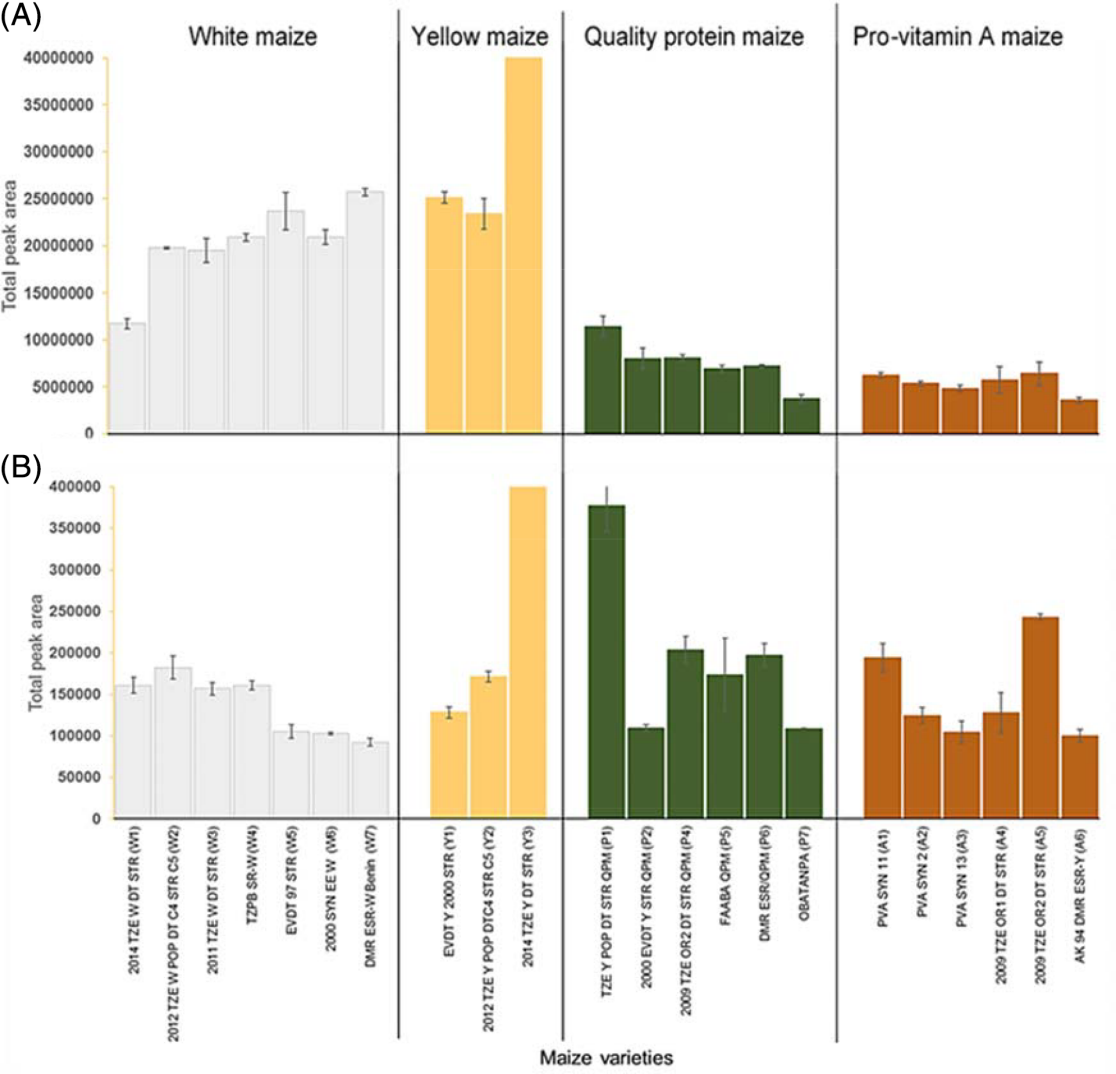
Abundance of volatile organic compound detected in maize flour (A) and their porridges (B) expressed as peak area, obtained as average of triplicate for flour and duplicate for porridge. Variety 2014 TZE Y DT STR (Y3) oversaturates the chart with total peak area + standard error of 8.6 + 0.4 (×108, counts*min) in flour and 5.3 + 0.08 (×105, counts*min) in porridge.

Figure 3 shows the abundance of VOCs in maize flour and porridge expressed as the average of total peak area of maize flour and porridge. The abundance of VOCs in the flour is in the order of yellow maize > white maize > QPM > PVA maize. In the porridges, a significant reduction was observed. This is in agreement with the PTR‐QiTOF‐MS results but in contrast to Zhang *et al*.,^7^ who observed a higher VOC concentration for millet after porridge preparation. However, in the aforementioned research, porridge was prepared in sealed vials thus measuring concentrated volatiles in the headspace. In this study, porridge preparation was based on the traditional African method, in which the escape of volatiles obviously occurs. In other research, Liu *et al*.^20^ observed that the content of volatile compounds in millet porridge decreased with an increase in water content. Apart from volatile loss by vaporization, a lower volatile composition of the porridge can be explained by the possibility of interaction of volatiles with the gelatinized maize matrix, that is volatiles trapped in the gel, thus resulting in less release to the headspace. Aromas form strong supramolecular interactions with gelatinized starches, thus increasing their retention in the matrix.^21^

Comparing the four groups of maize, the release of volatiles was more prominent in variety P1 and A5, mainly caused by hexanal and 1,2,4‐trimethyl benzene (psi‐cumene), respectively. High concentrations of trimethyl benzene have been reported in barley, oat, rye and wheat.^22^ Both hexanal and trimethyl benzene have also been linked to fungal volatiles and wheat off odour.^23^ The oversaturated peaks (bar chart in Fig. 3, not instrumental) shown for variety Y3 in maize flour and porridge emanate mainly from compounds such as 1‐ethyl‐3‐methylbenzene, 1,3,4‐trimethylbenzene and 1,2,4‐trimethylbenzene. These compounds have been reported to be present in cereal grains.^5^, ^22^ Trimethylbenzene derivatives were found to increase during heating of carotenoid‐rich sweet corn juice.^24^ Aroma compounds having a benzene ring have been proposed to be degradation products of carotenoids.^24^ Yellow and provitamin maize rich in carotenoid showed a significant increase in these compounds during thermal treatment of porridge. Zepka *et al*.^25^ found similar volatile compounds during thermal degradation of the carotenoids in cashew apple products.

**Figure 4:**
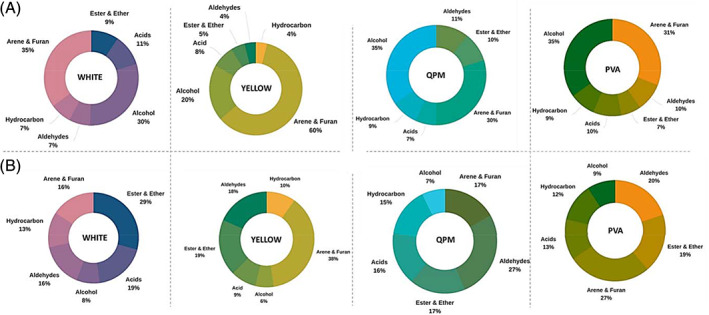
Volatile composition of maize flour (A) and porridge (B) identified by SPME‐GC–MS in white maize, yellow maize, quality protein maize (QPM) and provitamin A (PVA) maize. The bar charts represent the percentage total peak area of the average of each groups – white (*n* = 7), yellow (*n* = 3) QPM (*n* = 6) and PVA (*n* = 6).

The identified VOCs in the four maize groups (averages of all varieties in the same group) are shown in Tables 2 and 3. For maize flours, 48 VOCs were identified, namely saturated hydrocarbons (two); unsaturated hydrocarbons (one); acids (eight); alcohols (14); aldehydes (seven); sulphur‐containing compounds, esters and ethers (five); arenes and furans (11). The maize porridges generated a total of 21 VOCs, namely saturated hydrocarbons (four); acids (seven); alcohols (two); aldehydes (two); arenes and furans (four) and nitrogen‐containing compounds, esters and ethers (two). Figure 4 shows the volatile fractions in the flours and porridges according to chemical families. The charts also highlight the compositional changes that occur due to the transformation of flour into porridge. The two hydrocarbons – hexane and nonane identified in maize flour had relative total peak areas (%) of 7.08 ± 1.64, 4.16 ± 1.85, 8.62 ± 1.91 and 8.4 ± 2.15 for white, yellow, QPM and PVA, respectively (Table 2). Preparation of porridge generated decane, 2,6‐dimethylheptadecane, 6‐methyloctadecane and dodecane. No significant difference was found in hydrocarbons in maize flour and porridge (*P* > 0.05). Macku *et al*.^26^ reported similar GC–MS peak areas (%) in heated corn oil; octane and heptane were found to be the second and third most abundant peak area. Although hydrocarbons had high peak areas, their contribution to overall flavour may be low due to high human sensing thresholds.^8^ Limonene that has a very low flavour threshold value of 0.21 ppm (in water) was found to have between 0.19% and 0.33% peak area but its sensory contribution may be more significant.^8^

**Table 2:** Volatile organic compounds identified in maize flour of four variety groups: white maize, yellow maize, quality protein maize (QPM) and provitamin A (PVA) maize by headspace solid‐phase microextraction gas chromatography–mass spectrometry (HS‐SPME GC–MS) (extraction temperature 40 °C for 10 min)

Compounds	Maize porridge (average peak area % ± standard error)				*P*‐Value^a^
	White maize	Yellow maize	QPM	PVA maize	
*Hydrocarbon*					
Hexane	2.42 ± 0.9	1.8 ± 0.42	5.13 ± 1.24	4.18 ± 1.05	0.22
Nonane	4.66 ± 0.74	2.36 ± 1.43	3.49 ± 0.67	4.22 ± 1.1	0.44
Subtotal	7.08 ± 1.64	4.16 ± 1.85	8.62 ± 1.91	8.4 ± 2.15	
*Terpenes*					
Limonene	0.33 ± 0.04	0.25 ± 0.13	0.2 ± 0.03	0.19 ± 0.06	0.33
Subtotal	0.33 ± 0.04	0.25 ± 0.13	0.2 ± 0.03	0.19 ± 0.06	
*Aldehydes*					
Hexanal	4.99 ± 0.28	2.77 ± 1.32	8.76 ± 1.3	7.77 ± 1.13	**0.01**
Nonanal	0.7 ± 0.07	0.47 ± 0.2	0.65 ± 0.08	0.73 ± 0.14	0.72
2‐Undecenal	0.21 ± 0.01	0.13 ± 0.05	0.04 ± 0	0.02 ± 0	**0.01**
2,4‐Decadienal	0.46 ± 0.02	0.51 ± 0.23	0.39 ± 0.05	0.52 ± 0.05	0.38
2‐Butyl‐2‐octenal	0.15 ± 0.01	0.09 ± 0.01	0.12 ± 0.05	0.08 ± 0.02	0.64
3,4‐Dimethylbenzaldehyde	0.2 ± 0.02	0.13 ± 0.06	0.1 ± 0.02	0.05 ± 0.01	**0.01**
Benzaldehyde	0.6 ± 0.1	0.28 ± 0.13	0.57 ± 0.07	0.38 ± 0.05	0.08
Subtotal	7.31 ± 0.51	4.38 ± 2.00	10.63 ± 1.57	9.55 ± 1.4	
*Alcohols*					
2‐Butyloctanol	2.13 ± 0.18	1.54 ± 0.67	2.54 ± 0.45	2.35 ± 0.42	0.55
2‐Methyl‐1‐undecanol	2.12 ± 0.2	1.54 ± 0.68	2.15 ± 0.33	1.91 ± 0.2	0.67
1‐Methoxy‐2‐propanol	4.19 ± 0.74	1.9 ± 0.7	2.89 ± 0.89	2.49 ± 0.31	0.28
1‐Pentanol	1.79 ± 0.37	1.6 ± 0.83	2.44 ± 0.5	3.26 ± 0.48	0.18
1‐Octen‐3‐ol	1.08 ± 0.23	0.85 ± 0.4	0.91 ± 0.16	1.3 ± 0.17	0.48
2‐Ethylhexanol	0.4 ± 0.03	0.24 ± 0.11	0.22 ± 0.04	0.2 ± 0.02	**0.02**
1‐Nonanol	0.22 ± 0.02	0.16 ± 0.08	0.11 ± 0.02	0.15 ± 0.02	**0.03**
3‐Methoxybutanol	2.95 ± 0.84	1.52 ± 0.72	1.16 ± 0.19	1.06 ± 0.11	0.27
1‐Hexanol	4.22 ± 0.66	4.23 ± 2.02	5.84 ± 1.67	5.67 ± 0.7	0.56
Ethanol	6.36 ± 1.27	4.08 ± 1.89	14.11 ± 3.77	13.97 ± 2.07	
2‐Heptanol	0.58 ± 0.14	0.35 ± 0.17	0.59 ± 0.11	0.63 ± 0.09	0.70
Benzenemethanol	0.06 ± 0	0.05 ± 0.01	0.02 ± 0	0.03 ± 0.01	**0.01**
Mercaptoethanol	1.13 ± 0.05	0.94 ± 0.41	1.58 ± 0.14	1.95 ± 0.17	**0.00**
2,3‐Butanediol	3.26 ± 1.38	0.73 ± 0.64	0.15 ± 0.02	0.13 ± 0.02	**0.04**
Subtotal	30.49 ± 6.11	19.73 ± 9.33	34.71 ± 8.29	35.1 ± 4.79	
*Arenes and furans*					
Propylbenzene	0.13 ± 0.02	1.56 ± 1.13	0.55 ± 0.24	0.87 ± 0.29	0.07
2‐Pentylfuran	2.24 ± 0.54	1.54 ± 0.73	2.91 ± 0.59	3.72 ± 0.6	0.20
1,3,4‐Trimethylbenzene	0.19 ± 0.05	6.21 ± 5.03	2 ± 0.97	2.78 ± 1.21	0.07
1‐Ethyl‐3‐methylbenzene	1.56 ± 0.15	22.83 ± 17.84	8.02 ± 3.62	12.32 ± 5.71	0.08
1,4‐Dimethyl‐2‐ethylbenzene	0.48 ± 0.08	0.3 ± 0.16	0.2 ± 0.03	0.17 ± 0.03	**0.03**
1,2,3‐Trimethyl benzene	0.21 ± 0.05	4.32 ± 3.45	2.08 ± 0.89	2.29 ± 1.06	**0.02**
Indane	0.06 ± 0.01	0.98 ± 0.78	0.63 ± 0.26	0.34 ± 0.13	**0.01**
1,3‐Di‐*tert*‐butylbenzene	23.81 ± 2.66	16.18 ± 7.13	13.86 ± 1.7	8.12 ± 0.7	**0.01**
Durene	0.14 ± 0.02	0.13 ± 0.03	0.22 ± 0.07	0.18 ± 0.03	0.66
Benzocyclohexane	6.31 ± 1.07	5.93 ± 1.22	0.04 ± 0.01	0.04 ± 0.02	**0.02**
Phenol	0.24 ± 0.02	0.16 ± 0.06	0.18 ± 0.02	0.2 ± 0.02	0.20
Subtotal	35.37 ± 4.67	60.14 ± 37.56	30.69 ± 8.4	31.03 ± 9.8	
*Acids*					
Heptanoic acid	0.32 ± 0.01	0.22 ± 0.1	0.13 ± 0	0.13 ± 0.01	**0.02**
Octanoic acid	0.28 ± 0.01	0.19 ± 0.09	0.14 ± 0.01	0.11 ± 0.01	**0.01**
Hexanoic acid	1.36 ± 0.11	1.18 ± 0.54	1.02 ± 0.12	1.37 ± 0.14	0.23
Pentanoic acid	0.51 ± 0.03	0.39 ± 0.17	0.31 ± 0.03	0.36 ± 0.04	**0.04**
Butanoic acid	0.14 ± 0.02	0.21 ± 0.1	0.22 ± 0.07	0.54 ± 0.09	**0.04**
Acetic acid	7.79 ± 1.3	5.59 ± 2.56	5.04 ± 0.85	6.73 ± 0.96	0.60
Dimethyl ester butanedioic acid	0.23 ± 0.05	0.1 ± 0.05	0.08 ± 0.02	0.08 ± 0.01	**0.03**
4‐Hydroxybutanoic acid	0.26 ± 0.05	0.15 ± 0.07	0.14 ± 0.03	0.24 ± 0.03	0.18
Subtotal	10.89 ± 1.58	8.03 ± 3.68	7.08 ± 1.13	9.56 ± 1.29	
*Sulphur‐containing compounds, esters and ethers*					
1‐Propenethiol	6.42 ± 0.64	3.56 ± 1.51	8.44 ± 0.41	5.47 ± 0.16	
Sulphurous acid, cyclohexylmethyl hexyl ester	0.72 ± 0.02	0.44 ± 0.19	0.15 ± 0.01	0.13 ± 0.01	**0.005**
Dimethyl sulphone	0.16 ± 0.02	0.12 ± 0.05	0.13 ± 0.02	0.17 ± 0.02	0.551
Sulphurous acid, cyclohexylmethyl dodecyl ester	1.85 ± 0.08	1.16 ± 0.5	0.67 ± 0.15	0.36 ± 0.04	**0.006**
Vanillin, *tert*‐butyldimethylsilyl ether	0.31 ± 0.02	0.2 ± 0.09	0.48 ± 0.04	0.54 ± 0.05	**0.002**
Subtotal	9.46 ± 0.78	5.48 ± 2.34	9.87 ± 0.63	6.67 ± 0.28	

^a^ The *P*‐values (Kruskal–Wallis). *P* values marked in bold fonts are significantly different (*P* < 0.05).

**Table 3:** Volatile organic compounds identified in maize porridge of four variety groups: white maize, yellow maize, quality protein maize (QPM) and provitamin A (PVA) maize by headspace solid‐phase microextraction gas chromatography–mass spectrometry (HS‐SPME GC–MS) (extraction temperature 40 °C for 10 min)

Compounds	Maize porridge (average peak area % ± standard error)				*P*‐Value^a^
	White maize	Yellow maize	QPM	PVA maize	
*Saturated hydrocarbons*					
Decane	5.25 ± 1.58	4.62 ± 3.19	6.66 ± 1.79	4.37 ± 0.73	0.77
2,6‐Dimethylheptadecane	1.31 ± 0.47	1 ± 0.74	1.97 ± 0.75	2.05 ± 0.39	0.48
6‐Methyloctadecane	3.96 ± 0.85	2.59 ± 1.58	3.51 ± 1.24	2.93 ± 0.46	0.75
Dodecane	2.78 ± 0.66	1.61 ± 0.5	3.38 ± 0.61	3.05 ± 0.51	0.21
Subtotal	12.73 ± 3.56	9.82 ± 6.01	15.52 ± 4.39	12.4 ± 2.09	
*Acids*					
Acetic acid	10.5 ± 1.26	4.06 ± 1.17	12.16 ± 3.64	9.21 ± 1.33	0.12
2‐Methylpropanoic acid	4.24 ± 0.31	2.48 ± 0.7	2.5 ± 0.4	2.42 ± 0.29	**0.02**
3‐Hydroxydodecanoic acid	0.32 ± 0.03	0.22 ± 0.04	0.17 ± 0.1	0.13 ± 0.05	0.12
Hexanoic acid	2.09 ± 0.31	0.87 ± 0.21	0.95 ± 0.25	0.77 ± 0.13	**0.02**
Heptanoic acid	0.83 ± 0.06	0.49 ± 0.1	0.31 ± 0.09	0.32 ± 0.04	**0.01**
Octanoic acid	0.89 ± 0.07	0.51 ± 0.12	0.32 ± 0.09	0.3 ± 0.07	**0.01**
Subtotal	18.87 ± 2.04	8.63 ± 2.34	16.41 ± 4.57	13.15 ± 1.91	
*Alcohol*					
1‐Pentanol	5.32 ± 0.64	4.2 ± 1.45	5.12 ± 0.51	6.79 ± 0.84	0.27
2‐(2‐Ethoxyethoxy)ethanol	3.16 ± 0.28	2.01 ± 0.51	2.17 ± 0.32	2.4 ± 0.25	0.10
Subtotal	8.48 ± 0.92	6.21 ± 1.96	7.29 ± 0.83	9.19 ± 1.09	
*Aldehydes and ketones*					
2‐Heptanone	10 ± 1.52	3.64 ± 1.4	7.18 ± 1.19	12.65 ± 1.89	**0.02**
Hexanal	5.73 ± 2.59	14.75 ± 6.03	19.98 ± 8.74	7.42 ± 1.54	0.10
Subtotal	15.73 ± 4.11	18.39 ± 7.43	27.16 ± 9.93	20.07 ± 3.43	
*Arene and furan*					
2‐Methylfuran	4.35 ± 0.65	4.84 ± 2.12	2.65 ± 0.54	7.29 ± 1.58	**0.04**
2‐Pentylfuran	5.93 ± 0.76	3.76 ± 1.11	5.36 ± 0.8	6.29 ± 0.76	0.31
1,2,4‐Trimethylbenzene	0.42 ± 0.16	24.68 ± 23.28	1.9 ± 1.42	8.77 ± 5.25	0.10
1,3‐Ditertiarybutylbenzene	5.28 ± 2.14	4.55 ± 3.33	6.95 ± 2.2	4.64 ± 1.13	0.73
Subtotal	15.98 ± 3.71	37.83 ± 29.84	16.86 ± 4.96	26.99 ± 8.72	
*Nitrogen/sulphur containing compounds, esters and ethers*					
Acetonitrile	6.07 ± 0.55	4.09 ± 1	3.33 ± 1.29	1.8 ± 0.34	**0.01**
Mercaptoacetic acid, 2TMS derivative	21.98 ± 1.87	14.09 ± 4.15	13.39 ± 2.25	14.73 ± 1.53	**0.05**
Octyl ether	1.49 ± 0.17	0.95 ± 0.21	0.72 ± 0.21	2.56 ± 1.89	0.10
Subtotal	29.54 ± 2.59	19.13 ± 5.36	17.44 ± 3.75	19.09 ± 3.76	

^a^ The *P*‐values (Kruskal–Wallis). *P* values marked in bold fonts are significantly different (*P* < 0.05).

VOCs in the acid group are mainly responsible for cheesy, fatty, sour, vinegar, rancid, fruity and sweaty aroma.^8^ As shown in Table 2, the relative total peak area of the acids in maize flour of white, yellow, QPM and PVA were 10.89 ± 1.58, 8.03 ± 3.68, 7.08 ± 1.13 and 9.56 ± 1.29, respectively. Higher relative total peak areas were observed in maize porridge, that is: white, 18.87 ± 2.04; yellow, 8.63 ± 2.34; QPM, 16.33 ± 4.56; PVA, 13.15 ± 1.91 (see Fig. 4). The relative increase in acids from flour to porridge could be due to changes in other volatiles. Some compounds were lost during porridge preparation, thereby allowing the relative amount of acids to rise since they showed better stability. Although Goicoechea *et al*.^17^ reported that the number and proportion of acids (especially formic, acetic and hexanoic acids) increased in maize oil after a long storage (1–10 years) due to oxidation, this cannot be the case in this research since the analysis was after storing at 4 °C. However, it is known that in the mechanism of hexose fragmentation through 1‐deoxy‐2,4‐hexodiulose, hydrolytic beta‐dicarbonyl cleavage can lead to the production of acetic acid.^27^ Therefore, hexose reaction (in the presence of water, independent of oxygen) can explain acetic acid formed during porridge preparation. Acetic acid was found to have the highest relative peak area within the acid group. In maize flour, the white maize had the highest peak area for acetic acid, that is 7.79 ± 1.3, while QPM had highest peak area (12.16 ± 3.64) in porridge, respectively, see Table 3. Acetic acid has a strong, pungent, sour and vinegar odour but the flavour threshold value is high. Hence it likely contributes little to the flavour of maize. Other acids, for example butanoic acid, pentanoic acid, hexanoic acid, heptanoic acid and octanoic acid, have been reported to have extremely low flavour threshold values and hence their flavour impact is not negligible regardless of their infinitesimal amounts.^8^

Alcohols are important contributors to the overall green, floral, fruity, grassy, and earthy aroma profile of maize.^3^ Some members of this group possess extremely low threshold values (in water). For instance, 3‐hexan‐1‐ol has a flavour threshold value of 0.0038 ppm (in water); ethanol, which has the highest peak area in this research, has 53 ppm (in water).^8^ In total 14 alcohol compounds were detected in flour samples and two in porridge. From the highest to lowest, the alcohol contents in maize samples were: yellow, 19.73 ± 9.33; white, 30.49 ± 6.11; QPM, 34.71 ± 8.29; PVA, 35.1 ± 4.79, see Fig. 4. Furthermore, 1‐hexanol had the second‐highest percentage in flour while 1‐pentanol was the highest in porridge. Similar alcohol compositions were recently reported in gluten‐free flour and maize starch.^28^ Some of the alcohols detected, for example 1‐octen‐3‐ol, 1‐pentanol, 1‐nonanol, 2‐heptanol and 1‐hexanol, have been identified as the volatile compounds in cereals contributing to the green, grass‐like, mushroom, fruity, herbaceous, plastic, and citrus aroma characteristics.^11^, ^16^ In porridge these compounds were lost, leaving only 1‐pentanol. Alcohols in food can be abundant but their impact on flavour is likely to be lower than that of aldehydes.^8^

Aldehydes are very prominent in VOC composition of food and their flavour threshold level for human sensing is low, implying crucial contributions to aroma perception. Aldehydes mainly contribute green, grassy, soapy, citric and malty notes.^8^ In maize flour, aldehyde peak areas (%) were: white, 7.31 ± 0.51; yellow, 4.38 ± 2.00; QPM, 10.63 ± 1.57; PVA, 9.55 ± 1.4, see Fig. 4. The number of different aldehydes was reduced from seven in maize flour to only hexanal in porridge. Hexanal is very common in food aroma, and when considered in a single state, has a green bean and grassy character. Pyysalo *et al*.^29^ observed a low odour threshold value for hexanal, that is 0.02 (mg L^−1^ in water), signifying its important contribution to the overall aroma of maize. The proportion of hexanal was most prominent in PVA maize and QPM. Hexanal is commonly formed during cooking processes, thus the higher proportion in the porridge was to be expected. Hexanal can be generated by lipid breakdown (commonly oxidative degradation of linoleic acid) initiated by lipoxygenase.^5^, ^17^ In fact, hexanal is often used as an indicator for human perception of rancidity. Other researchers have found hexanal to be most abundant in maize starch.^5^ The high proportion of hexanal and 2‐heptanone in carotenoid‐rich maize could be attributed to enzymatic degradation of the oil. Besides, autoxidation of free fatty acids has been described as a major source of ketones by Mottram and Maarse ^8^ but only 2‐heptanone was found in this research. Two‐heptanone (having a fruity, green, nutty, soapy smell) has been identified as an odour‐active compound in maize, thus its contribution to maize aroma is relevant.^3^, ^5^

Aromatic hydrocarbons (arene) and furans are interesting groups of VOCs in maize. In maize flour, the peak areas (%) were: white, 35.37 ± 4.67; yellow, 60.14 ± 37.56; QPM, 30.69 ± 8.4; PVA, 31.03 ± 9.8, see Fig. 4 and Table 3. Most VOCs of the yellow maize variety belonged to this group; this may be an important classification characteristic of the variety. Furans can potently contribute to aroma, even in minute amounts. Two main furans were found in our samples: 2‐methyl furan and 2‐pentyl furan. The latter is known for its crucial contribution to the aroma and flavour of popcorn.^6^ Furans are usually formed during thermal processing of food, thus explaining the increase in porridge. Benzene derivatives such as propyl benzene, 1‐ethyl‐3‐methylbenzene and 1,3,4‐trimethyl benzene were found to be a major contributor of VOCs in maize, especially for the yellow maize cultivar. Aromatic hydrocarbons and furans have relatively low threshold values. Hence their presence as found in all the samples especially the carotenoid‐rich varieties is noteworthy for their role in maize aroma.

## CONCLUSIONS

This is the first study on volatile compounds in maize using PTR‐QiTOF‐MS. Maize varieties from different groups were successfully grouped by PCA. With such sophisticated, highly sensitive and fast equipment, coupled with a statistical tool for multivariate analysis, the diversity in maize odour‐active compounds can be assessed as an input to improve sensory qualities of new varieties. HS‐SPME GC–MS was used to identify VOCs and this was also the first time the method was used on improved maize cultivars developed for the nutritional needs of the growing African population. The study identified differences in volatile composition of maize cultivars and their corresponding porridges. Results of this study can be the basis for further research of flavour threshold values and identification of the association between consumer perception and the inherent volatiles compounds. This will contribute to the understanding of consumer preferences for maize varieties in Africa and help breeders to build a data bank for volatiles that will enable the incorporation of aroma traits during variety selection and development. Breeding for aroma is a difficult task for maize breeders because aroma is yet to be properly understood and classified genetically, but our current research paves ways to generate information useful for further classification. Our results show that maize has a vast volatile variability that is still untapped: considering that aroma is a major contributor to taste and food crop acceptance, this research aspect requires further efforts. An investigation into methods to monitor off‐flavour during post‐harvest handling of the nutrient‐rich maize varieties is recommended. For instance, hexanal production is a good indicator of lipid oxidation and could be used to evaluate storage stability, especially in provitamin A biofortified maize.

## Supporting information


**Table S1.** Overview of the maize varieties (*n* = 22) used in this study.

**Table S2.** Supporting Information

**Table S3.** Supporting Information
