# Supplementary material for: Identification of the volatile profiles of 22 traditional and newly bred maize varieties and their porridges by PTR‐QiTOF‐MS and HS‐SPME GC–MS
Source: J Sci Food Agric. 2020 Sep 21;101(4):1618–28. doi: 10.1002/jsfa.10781 (PMC7891407; doi:10.1002/jsfa.10781)
Supplement: Supplementary file 1 — Table S1. Overview of the maize varieties (n = 22) used in this study. [file JSFA-101-1618-s001.docx]

Table S1: Overview of the maize varieties (n = 22) used in this study.

| Group | n | Varieties (abbreviated codes in brackets) | Description | Appearance |
| --- | --- | --- | --- | --- |
| Pro-vitamin A biofortified maize | 6 | PVA SYN 11 (A1); PVA SYN 2 (A2); PVA SYN 13 (A3); 2009 TZE OR1 DT STR (A4); 2009 TZE OR2 DT STR (A5); AK 94 DMR ESR-Y (A6) | High in provitamin A, pest resistance and high yield varieties. | Orange - yellow endosperm colour. |
| Quality protein maize | 6 | TZE Y POP DT STR QPM (P1 - yellow); 2000 EVDT Y STR QPM (P2 - yellow); 2009 TZE OR2 DT STR QPM (P4 - yellow); FAABA QPM (P5 - white); DMR ESR/QPM (P6 - white); OBATANPA (P7 - white) | Drought tolerant (except P2), resistance to striga infestation, early maturity. Higher in lysine and tryptophan (P5 and P6). | White endosperm colour (P3, P5, P6 and P7). Yellow endosperm colour (P1, P2). Orange endosperm colour (P4). |
| White maize | 7 | 2014 TZE W DT STR (W1); 2012 TZE W POP DT C4 STR C5 (W2); 2011 TZE W DT STR (W3); **TZPB SR-W (W4)**; EVDT 97 STR (W5); 2000 SYN EE W (W6); **DMR ESR-W (W7)** | Drought tolerant, resistance to striga infestation and early maturity (W2, W3 and W7). Drought tolerance, increased yield and pest resistance (W4, W5, W6). **DMR ESR-W is high yield dent released in 1987 while TZPB SR-W was developed in 1970.** | White endosperm colour and normal endosperm type.  Glassy and floury (W5, W6 and W7). Slightly bigger kernels (W4). |
| Yellow maize | 3 | EVDT Y 2000 STR (Y1); 2012 TZE Y POP DTC4 STR C5 (Y2); 2014 TZE Y DT STR (Y3) | Drought tolerant (except Y1), resistance to striga infestation, early maturity. | Yellow endosperm colour and normal endosperm type. |

(n) Number of maize varieties per group
